# PJ34 prevents cisplatin-induced hair cell loss via inhibition of PARP-1–AIF parthanatos

**DOI:** 10.17305/bb.2025.12533

**Published:** 2025-06-20

**Authors:** Huiming Nong, Xiru Zhang, Yingxue Yuan, Junhong Zhang, Jingyi Zhao, Zhixin Cao

**Affiliations:** 1 Department of Otolaryngology, Shandong Provincial Hospital Affiliated to Shandong First Medical University, Shandong Province, China; 2 Department of Pathology, Shandong Provincial Hospital Affiliated to Shandong First Medical University, Shandong Province, China

**Keywords:** Hair cells, HC, hearing loss, cisplatin, PARP inhibitor, apoptosis-inducing factor, AIF, parthanatos

## Abstract

The poly (ADP-ribose) polymerase-1 (PARP-1) inhibitor PJ34 acts as an anti-inflammatory and neuroprotective agent by modulating parthanatos. This study aimed to explore the protective effects of PJ34 against cisplatin-induced injury in auditory cells and to elucidate its underlying mechanism of action. Flow cytometry and immunofluorescence were employed to detect apoptosis in HEI-OC1 and ovarian cancer cell lines. Additionally, immunofluorescence and Western blotting were used to assess changes in the expression of related proteins, including cleaved Caspase-3, PARP-1, and cytosolic apoptosis-inducing factor (AIF), across the groups. Mitochondrial membrane potential (MMP) levels were measured using the MMP assays, and reactive oxygen species (ROS) levels were assessed by MitoSox red staining. Our results indicate that treatment with 30 µM cisplatin activates cleaved Caspase-3, promotes PARP-1 overexpression, and facilitates AIF nuclear translocation, leading to decreased MMP and increased ROS accumulation, which ultimately triggers auditory cell death. Treatment with 2.5 µM PJ34 mitigated PARP-1 overexpression and AIF nuclear translocation following cisplatin exposure, reduced the decline in MMP, and decreased ROS accumulation, thereby alleviating damage to auditory cells. Conversely, PJ34 enhanced the damaging effects of cisplatin on ovarian cancer cell lines. In conclusion, our findings suggest that PJ34 may reduce cisplatin-induced hair cell death by regulating PARP-1-mediated parthanatos. Notably, PJ34 shows promise as a potential novel therapeutic agent for the prevention and/or treatment of cisplatin-induced ototoxicity.

## Introduction

Poly (ADP-ribose) polymerase-1 (PARP-1) is a 116 kDa multifunctional nuclear protease that regulates a variety of biological functions [1]. As part of a group of enzymes responsible for DNA damage monitoring and repair, PARP-1 is activated when cellular DNA is damaged, initiating the DNA repair mechanism through PARylation. Interestingly, the degree of PARP-1 activation depends on the extent of DNA lesions. Hyperactivation of PARP-1 can cause nuclear translocation of apoptosis-inducing factor (AIF), which, in turn, triggers parthanatos, contributing to cell death [2].

In many pathological conditions, oxidative-dependent damage leads to extensive DNA breaks, hyperactivating PARP-1. The nucleus then produces and releases toxic levels of poly (ADP-ribose) (PAR) into the plasma, where PAR binds to mitochondrial membrane proteins. This leads to increased mitochondrial membrane permeability and depolarization, facilitating the mitochondrial release of AIF into the cytoplasm. AIF then binds to macrophage migration inhibitory factor (MIF) and enters the nucleus, resulting in chromatin and DNA fragmentation that triggers a PARP-1-mediated cell death program [3, 4]. Relevant studies have demonstrated that the PARP-1 inhibitor (PARPi) PJ34 can block PARP-1 binding to nicotinamide adenine dinucleotide (NAD+) through a competitive inhibition mechanism. This prevents NAD+ overconsumption and reduces the depletion of the cellular energy pool, thereby alleviating metabolic disorders and subsequent cell death [5, 6]. In recent years, studies have shown that PJ34 may exert a protective effect on the intestinal mucosal structure in mice with acute colitis and provide neuroprotective effects after traumatic brain injury [1, 7]. Furthermore, numerous studies have shown that PARP-1 expression is upregulated in cancer cell lines [8, 9]. Inhibiting PARP-1 leads to decreased DNA repair function and is considered an important mechanism for alleviating cisplatin resistance in cancer cells [10]. It has been shown that PJ34 can increase sensitivity to cisplatin therapy in non-small cell lung carcinoma (NSCLC) cellular models by increasing the accumulation of platinum adducts [11]. Additionally, PJ34 can work synergistically with gemcitabine and cisplatin to induce more cancer cell death in triple-negative breast cancer cells [12]. Many studies have demonstrated that PARPi not only exert synergistic anticancer effects with cisplatin but also reduce cisplatin-induced nephrotoxicity. Pharmacological inhibition or genetic ablation of PARP-1 in murine models of cisplatin-mediated nephrotoxicity has been reported to reduce renal histopathological damage and attenuate cisplatin-induced nephrotoxicity [13]. The combination of PJ34 and 3-aminobenzamide ameliorates renal function and histological damage in zebrafish and mouse models of cisplatin nephrotoxicity [14]. Given the variability in the effects of PJ34 and cisplatin under different pathological conditions, exploring the impact of PJ34 on cisplatin-induced ototoxicity is a rational research approach. Indeed, multiple studies have indicated that suppression of PARP-1 is a potentially effective strategy for attenuating cisplatin-induced ototoxicity. Overexpression of PARP-1 has been implicated in cisplatin-induced damage to cochlear tissues and HEI-OC1 cells, while suppression of PARP-1 activation has been shown to alleviate ototoxicity by restoring ATP and NAD+ levels decreased by cisplatin [15]. Another study showed that inhibition of PARP-1 expression and reduction of AIF nuclear translocation could attenuate cisplatin-induced stria vascularis damage, thereby reducing hearing loss [16]. In addition, PARP-1 inhibition has also been shown to reduce hearing loss induced by streptozotocin and noise exposure [17, 18]. Taken together, PARP-1 is considered a potential key target for the treatment of hearing impairment. Cisplatin, a platinum-based chemotherapeutic agent, remains a cornerstone in the clinical management of various human malignancies. However, its use is severely limited due to serious side effects, including nephrotoxicity and ototoxicity [19, 20]. Hearing loss of varying degrees has been reported in approximately 40%–60% of patients treated with cisplatin, with 18% developing severe to profound hearing loss [21]. The mechanism underlying cisplatin-induced hearing loss is not fully understood, but current studies suggest it is primarily due to increased reactive oxygen species (ROS) and mitochondrial dysfunction [21, 22]. Increased mitochondrial ROS levels following cisplatin exposure initiate a cascade that reduces mitochondrial membrane potential (MMP), inducing the mitochondrial apoptotic pathway mediated by Bcl-2 and cleaved caspase-3, resulting in irreversible auditory cell death [23]. Moreover, cisplatin has been reported to cause cochlear stria vascularis damage in mice through activation of cleaved caspase-3 and PARP-1, as well as promotion of AIF nuclear translocation, leading to severe ototoxicity [16].

To date, no studies have examined the effect of PJ34 on cochlear hair cell (HC) damage induced by cisplatin. Therefore, we investigated whether PJ34 could attenuate cisplatin-induced injury in mouse HCs and the HEI-OC1 cell line, with a particular focus on changes in PARP-1 and AIF expression, mitochondrial apoptotic pathway dynamics, and mitochondrial function. Furthermore, we used the same concentrations of PJ34 and cisplatin to co-treat tumor cell lines to assess whether PJ34 could mitigate cisplatin ototoxicity without compromising its tumoricidal effect. To evaluate the effect of PJ34 on the anticancer activity of cisplatin, we assessed the apoptotic rate of ovarian cancer TOV112D and HEY cell lines treated with different concentrations of PJ34 and 30 µM cisplatin using flow cytometry. If confirmed, PJ34 would be a promising pharmacological candidate for ameliorating cisplatin-induced ototoxicity and could become the first drug to achieve effective tumor cell killing while minimizing the adverse effects of cisplatin-induced ototoxicity and nephrotoxicity.

## Materials and methods

### Animals and treatments

This study utilized C57BL/6 mice, aged 3 days postpartum (P3). The mice were procured from an ISO-certified supplier (Spelford Biotechnology Co., Beijing, China) for experimental implementation. According to the AVMA (American Veterinary Medical Association), physical methods (e.g., cervical dislocation rapid amputation) are permissible for rodent pups ≤7 days old without anesthesia due to their immature nociceptive pathways. In this experiment, a pre-checked WPI mouse guillotine was used. P3 mice were gently grasped, manually immobilized, their heads positioned in the guillotine slot, and decapitated in a single attempt to ensure rapid interruption of cerebral blood supply and loss of consciousness. Death was confirmed by absence of heartbeat, respiration, and pupillary reflexes. Operator Huiming Nong is experienced and trained. The study was approved by the Animal Protection Committee of Shandong Provincial Hospital (Approval No. ECAESDUSM 20123011). Additionally, euthanasia protocols were referenced from Herling (2016) [24] and Kumar and Bansal (2017) [25].

### Cell line and cell culture

The HEI-OC1 auditory progenitor cell line (RRID: CVCL-D899, KMCC-001-1439, Coweldgen Scientific Co., LTD, CN), an *in vitro* model exhibiting phenotypic characteristics reminiscent of cochlear HCs and originating from murine organ of Corti explants, was maintained in DMEM-high glucose formulation (C11995500BT, Gibco, USA) supplemented with 10% (v/v) fetal bovine serum (FBS, 16000–044, Gibco, USA) under antibiotic-free conditions, following standardized culture protocols (33 ± 0.5 ^∘^C, 5% CO_2_, 95% humidity). HEY cell lines (RRID: CVCL-0671, CL-0671, Pricella, CN) were cultured in HEY medium (CM-0671, Pricella, CN). TOV112D cell lines (RRID: CVCL-3612, FH1271, FuHeng Biology, CN) were cultured in TOV112D medium (FH-TOV-112D, FuHeng Biology, CN). Both HEY and TOV112D cell lines were cultured under standard conditions (37 ^∘^C, 5% CO_2_). The HEI-OC1 cell line was identified using a real-time quantitative PCR detection system (qPCR), and HEY and TOV112D cell lines were identified by short tandem repeat (STR) profiling. All cell lines tested negative for mycoplasma.

### Cochlear explants culture and treatment

P3 C57BL/6 mice were decapitated, and the skull was dissected along the mid-cranial suture to expose the bilateral temporal bones. The cochleae were carefully extracted under a microscope. The entire basilar membrane was dissected and placed flat on a 10 mm thick slide pre-coated with Cell-Tak adhesive. Explants were cultured in Gibco™ DMEM/F12 Basal Medium (1056018, Gibco, USA) supplemented with B-27™ Serum-Free Supplement (17504044, Gibco, USA), N-2™ Supplement (17502048, Gibco, USA), and ampicillin sodium salt (A1170, Solarbio, China) at 37 ± 0.5 ^∘^C in a humidified incubator (5% CO_2_, 95% air) under sterile conditions.

### Drug treatments

Building upon previous research using 30 µM cisplatin (P4394, Sigma-Aldrich, USA) for 24-hour exposure [26], this study adopted the same cisplatin dosing protocol while assessing synergistic effects with PJ34 (HY-13688A, MedChemExpress, USA). Experimental groups included a cisplatin-only group and a combination treatment group receiving 2.5 µM PJ34 concurrently for 24 h.

### Immunofluorescence staining

After removing the original medium and washing three times with pre-cooled PBS, cells were fixed with 4% paraformaldehyde (PFA), permeabilized with Triton X-100/PBS solution, and blocked with 1% bovine serum albumin (BSA) in PBS (pH 7.4) for 1 hour at 22 ± 1 ^∘^C. Cells were then incubated overnight at 4 ^∘^C with primary antibody cocktails in a humidified chamber. The primary antibodies included anti-PARP-1 (1:250, 13371-1-AP, Proteintech, CN), anti-AIF (1:250, 17984-1-AP, Proteintech, CN), and anti-cleaved caspase-3 (1:500, 9664S, CST, US). After three PBS washes, samples were incubated with fluorescent secondary antibodies and DAPI (D9542, Sigma-Aldrich, US) for 1 hour in the dark. Slides were mounted and imaged using a Leica TCS SP8 confocal laser scanning microscope (Leica Microsystems, Germany) with 405/488/552/647 nm lasers and a 63× oil-immersion objective (NA 1.4). Images shown are representative of separate cochlear explants.

### MitoSox red staining

After treatment, cochlear explants were rinsed three times with PBS (pH 7.4) at 37 ^∘^C and incubated for 10 min with 3 µM MitoSox Red mitochondrial superoxide indicator (ThermoFisher, USA) under light-protected conditions at 22 ± 1 ^∘^C. Fluorescence was detected using a Leica TCS SP8 confocal fluorescence microscope (Leica Microsystems, Biberach, Germany).

### Protein extraction and Western blot (WB)

Total proteins were extracted using RIPA lysis buffer (R0020, Solarbio, CN) containing protease (P0100, Solarbio, CN) and phosphatase inhibitors (HY-K0022, MCE, CN). After centrifugation, the supernatant was collected and protein concentration determined using a BCA Protein Assay Kit (PC0020, Solarbio, CN). Proteins were separated via 10% SDS-PAGE and transferred onto PVDF membranes (0.45 µm, ISEQ00010, Merck Millipore, CN) for 90 min. Membranes were blocked with 5% BSA or 5% non-fat dry milk in TBST (Tris-buffered saline with 0.1% Tween-20) for 1 hour at 22 ± 1 ^∘^C. Membranes were then incubated with diluted primary antibodies for 1 hour at room temperature. Final detection was performed using the ECL Kit (WBKLS0100, Millipore, US), and results were analyzed with ImageJ software [27]. Primary antibodies used were anti-PARP-1 (1:1000), anti-AIF (1:1000), anti-cleaved caspase-3 (1:1000), and anti-GAPDH (1:5000).

### Flow cytometry

Apoptosis was assessed using the Annexin V/PI apoptosis detection kit (556547, BD Biosciences) and analyzed via flow cytometry on a FACS Calibur system (BD, US) using 10,000 cells per group. Data analysis was performed with FlowJo 10.8 software.

### Co-localization analysis of AIF and DAPI

Co-localization of AIF (red) and DAPI (blue) was analyzed using ImageJ software. A scale bar (20 µm) was calibrated, and a typical cell was selected. A line was drawn along the longitudinal axis using the “Line Segment” tool, and fluorescence intensity was measured using the “Plot Profile” tool. The x-axis represented distance (µm), and the y-axis indicated fluorescence intensity (a.u.). Overlap of the AIF and DAPI curves indicated co-localization.

### Cytoplasmic and nuclear protein extraction

Cytoplasmic and nuclear proteins were extracted using a Nuclear and Cytoplasmic Protein Extraction Kit (P0027, Beyotime Biotechnology, Shanghai, China). Adherent cells were collected from six-well plates with PBS, centrifuged, and the supernatant discarded. Pellets were resuspended in 200 µL of Reagent A with 1 mM PMSF (P0100, Solarbio, CN), incubated on ice for 15 min, followed by addition of 10 µL Reagent B. After centrifugation at 16,000 g at 4 ^∘^C for 5 min, the cytoplasmic protein-containing supernatant was collected. The nuclear pellet was resuspended in 50 µL of nuclear extraction reagent with 1 mM PMSF, vortexed, incubated on ice, and centrifuged again to collect nuclear proteins.

### Mitochondria membrane potential (MMP, Δ ψm)

MMP changes were measured using the JC-1 Mitochondrial Membrane Potential Assay Kit (Beyotime). Processed cells were washed twice with cold PBS and incubated in 500 µL culture medium and 500 µL JC-1 working solution at 37 ^∘^C for 20 min in the dark. Healthy cells with intact MMP accumulate JC-1 in mitochondria, forming red-emitting J-aggregates. In contrast, cells with disrupted MMP retain JC-1 in monomeric form in the cytoplasm, emitting green fluorescence.

### Cell counting

Images of cochlear tissue were acquired using a 40×2.5 objective. ImageJ software was used to quantify immunostained positive cells. The scale was calibrated to 40 µm using the scale bar in the image. The total length of the cochlear epithelium was measured, and the “Point Selections” tool was used to count immunostained cells. Quantification was expressed as cells per 0.1 mm, calculated by: (Total immunostaining-positive cells/ Total epithelial length in µm) × 100.

### Ethical statement

The research protocol was reviewed and approved by the Animal Protection Committee of Shandong Provincial Hospital affiliated with Shandong First Medical University (Approval No. ECAESDUSM 20123011).

### Statistical analysis

Data are presented as mean ± SD from at least three independent experiments. Statistical analyses were conducted using Microsoft Excel (2016) and SPSS 27 (27.0.1.0, IBM, US). One-way ANOVA followed by Dunnett’s post hoc test was used for comparisons involving more than two groups. Normality was tested using the Shapiro-Wilk test; homogeneity of variance was assessed using Levene’s test. When data violated normality assumptions, the Kruskal-Wallis *H*-test was applied. Welch’s ANOVA was used when variance homogeneity was not met. Dunnett’s test was preferred over Tukey’s HSD or Bonferroni correction when comparing multiple groups to a single control due to its better control of Type I errors and higher statistical power. A *P* value < 0.05 was considered statistically significant.

## Results

### PJ34 protects HEI-OC1 cells and HCs from cisplatin-induced cellular damage

**Figure 1.**
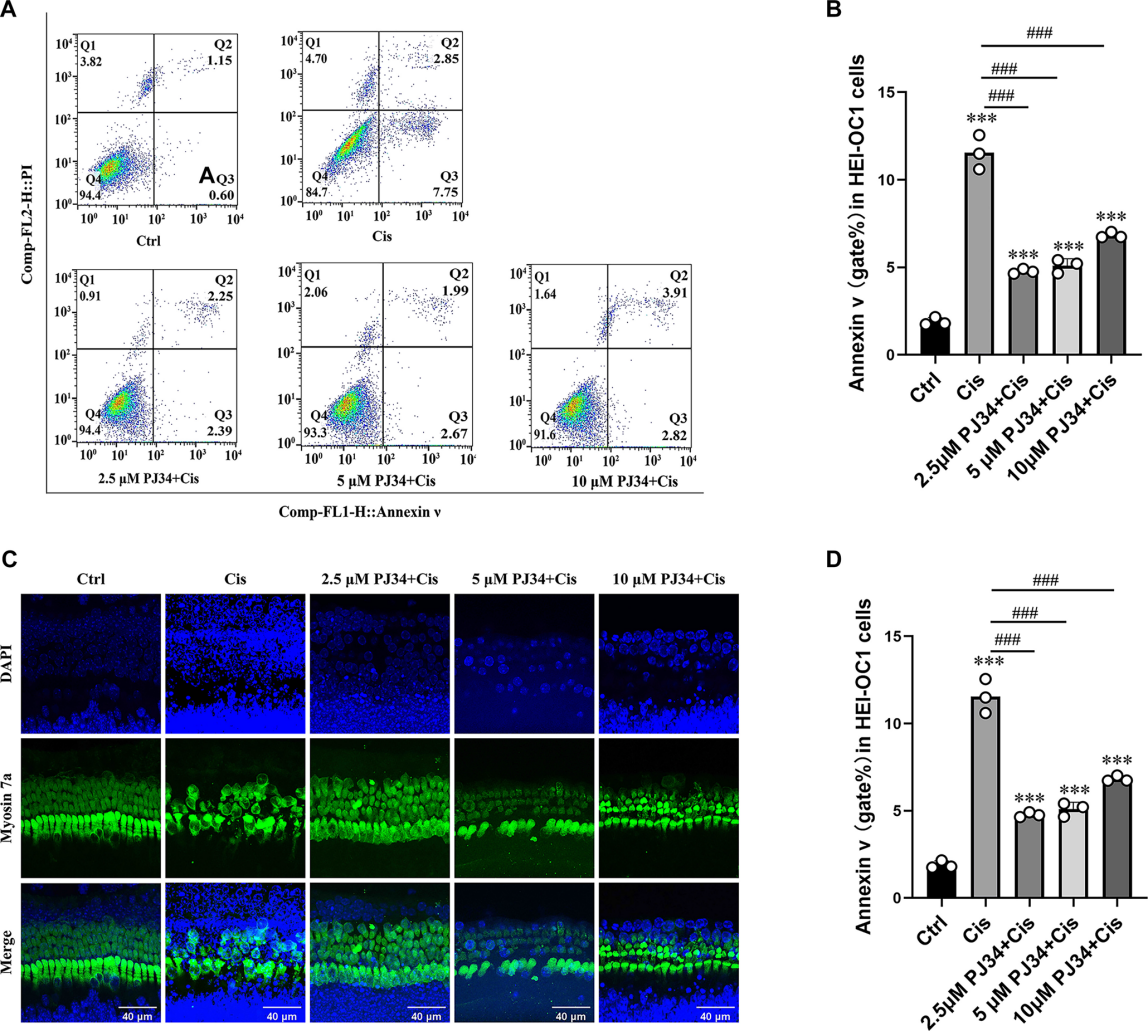
**PJ34 ameliorates cisplatin-induced injury in HEI-OC1 cells and HCs.** (A) Flow cytometry analysis of apoptosis in HEI-OC1 cells treated with various concentrations of PJ34 in the presence of 30 µM cisplatin; (B) Quantification of apoptotic rates shown in (A); (C) Immunofluorescence staining illustrating morphological changes in HCs treated with 30 µM cisplatin and varying concentrations of PJ34. HCs are labeled with Myosin 7a (green) and DAPI (blue). Representative images are shown for each group; (D) Quantification of Myosin 7a-positive HCs in each treatment group. ***P < 0.001 vs control group, ^##^*P* < 0.01 vs cisplatin group, ^###^*P* < 0.001 versus cisplatin group. *n* ═ 3. Scale bars ═ 40 µm. HCs: Hair cells.

Flow cytometry analysis demonstrated a marked elevation in the apoptotic index in the 30 µM cisplatin group (F(4,10) ═ 158.3; 6.10 ± 0.41 for Control; *P* < 0.001). In the PJ34 plus cisplatin group, the apoptosis rate was significantly reduced at a concentration of 2.5 µM PJ34 (0.41 ± 0.02 for Cisplatin; *P* < 0.001). As the concentration of PJ34 increased, the apoptosis rate gradually rose, indicating that 2.5 µM PJ34 was the most effective concentration in minimizing cisplatin-induced apoptosis in HEI-OC1 cells (Figure 1A and 1B).

Immunofluorescence analysis showed that HCs in the cisplatin group exhibited extensive degeneration and morphological abnormalities, including cytoplasmic atrophy, structural disorganization, and widespread detachment of HCs (F(4,10) ═ 59.57; 0.36 ± 0.04 for Control; *P* < 0.001). The PJ34 plus cisplatin group displayed more surviving HCs with fewer morphological alterations and better alignment. At 2.5 µM PJ34, the number of surviving HCs was highest, with four structurally complete rows of HCs (2.64 ± 0.34 for Cisplatin, *P* < 0.001). At 5 µM, some HCs were missing and cell arrangement was slightly disordered (1.91 ± 0.21 for Cisplatin; *P* < 0.001). At 10 µM, outer HCs were severely depleted, inner HCs exhibited morphological changes, and arrangement was further disrupted (1.57 ± 0.11 for Cisplatin; *P* < 0.01) (Figure 1C).

Cell counting confirmed a significant reduction in surviving HCs in the cisplatin group, which was attenuated by PJ34 co-treatment, especially at 2.5 µM PJ34 (Figure 1D). Based on these findings, 2.5 µM PJ34 was selected as the optimal concentration for subsequent experiments.

### PJ34 promotes apoptosis in cisplatin-treated ovarian cancer cell lines

**Figure 2.**
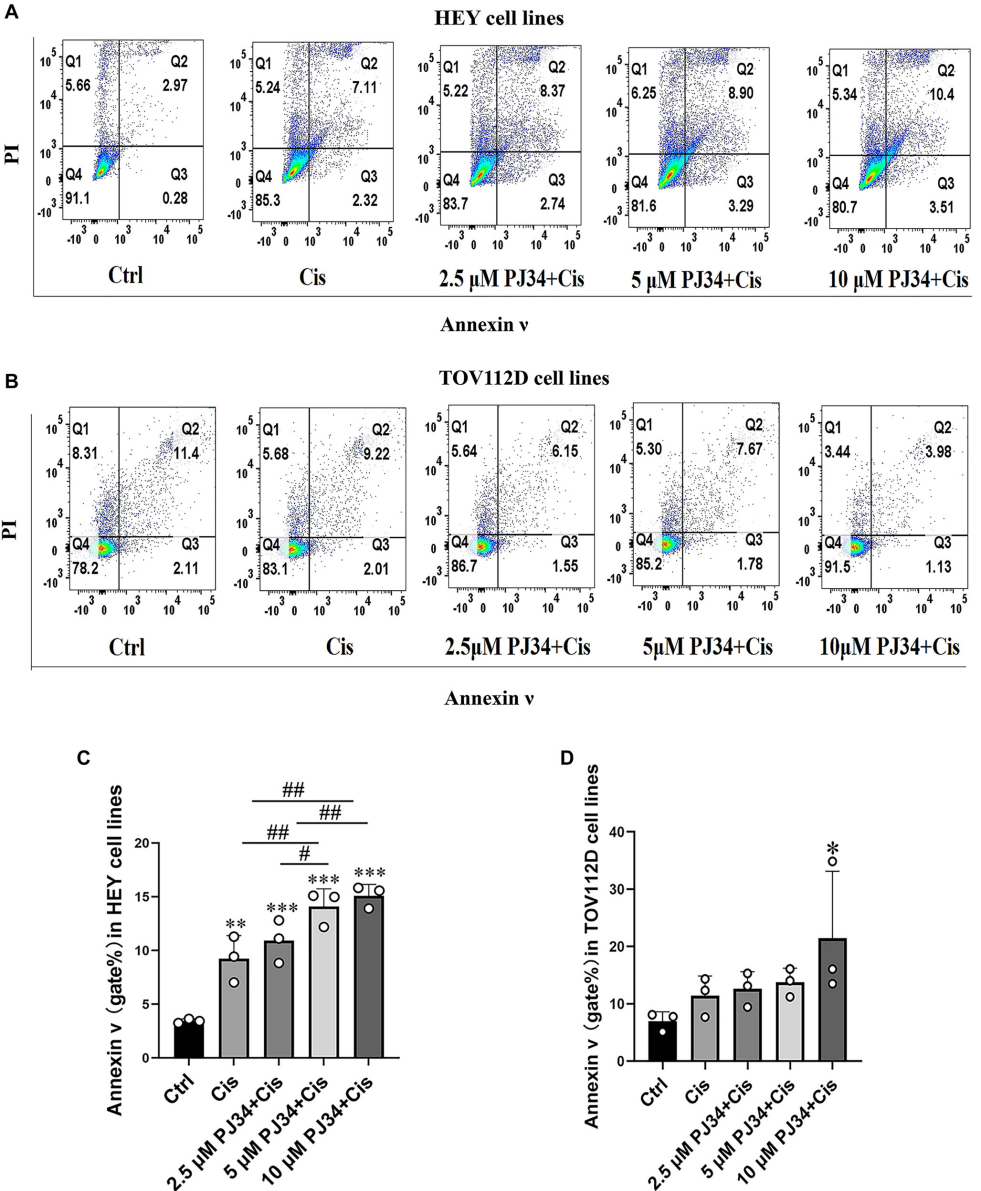
**PJ34 aggravates cisplatin-induced cell death in HEY and TOV112D cell lines.** (A and C) Apoptotic rate of HEY cells assessed by flow cytometry and corresponding quantitative analysis; (B and D) Apoptotic rate of TOV112D cells assessed by flow cytometry and corresponding quantitative analysis. *P< 0.05 vs control group, **P< 0.01 vs control group, ***P< 0.001 vs control group, ^#^*P*< 0.05 vs cisplatin group, ^##^*P*< 0.01 vs cisplatin group. A total of 10,000 cells were collected for each set of samples (*n* ═ 3).

Flow cytometry results indicated that treatment of HEY cell lines with 30 µM cisplatin significantly increased apoptosis compared to control (F(4,10) ═ 2; 9.25 ± 2.14 for Control, *P* < 0.01). When co-treated with PJ34, the apoptosis rate increased further compared to cisplatin alone (1.19 ± 0.06, *P* > 0.05 for 2.5 µM + Cisplatin; 1.59 ± 0.49, *P* < 0.01 for 5 µM + Cisplatin; 1.70 ± 0.45, *P* < 0.01 for 10 µM + Cisplatin). A positive correlation was observed between PJ34 concentration and apoptosis rate (Figure 2A and 2C).

In TOV112D cells, the apoptotic rate after 30 µM cisplatin treatment was slightly higher than in the control group but was not statistically significant (F(4,10) ═ 2.518; 1.62 ± 0.13 for Control; *P* > 0.05). Treatment with 2.5 µM and 5 µM PJ34 had a minimal effect on cisplatin-induced apoptosis. However, at 10 µM PJ34, the apoptosis rate increased significantly (21.48 ± 11.67 for Control; *P* < 0.05) (Figure 2B and 2D).

Overall, PJ34 enhanced the cytotoxic effects of cisplatin in both TOV112D and HEY cell lines, although the degree of enhancement varied by cell line and PJ34 concentration.

### PJ34 alleviates cell death following cisplatin action in a caspase-independent manner

**Figure 3.**
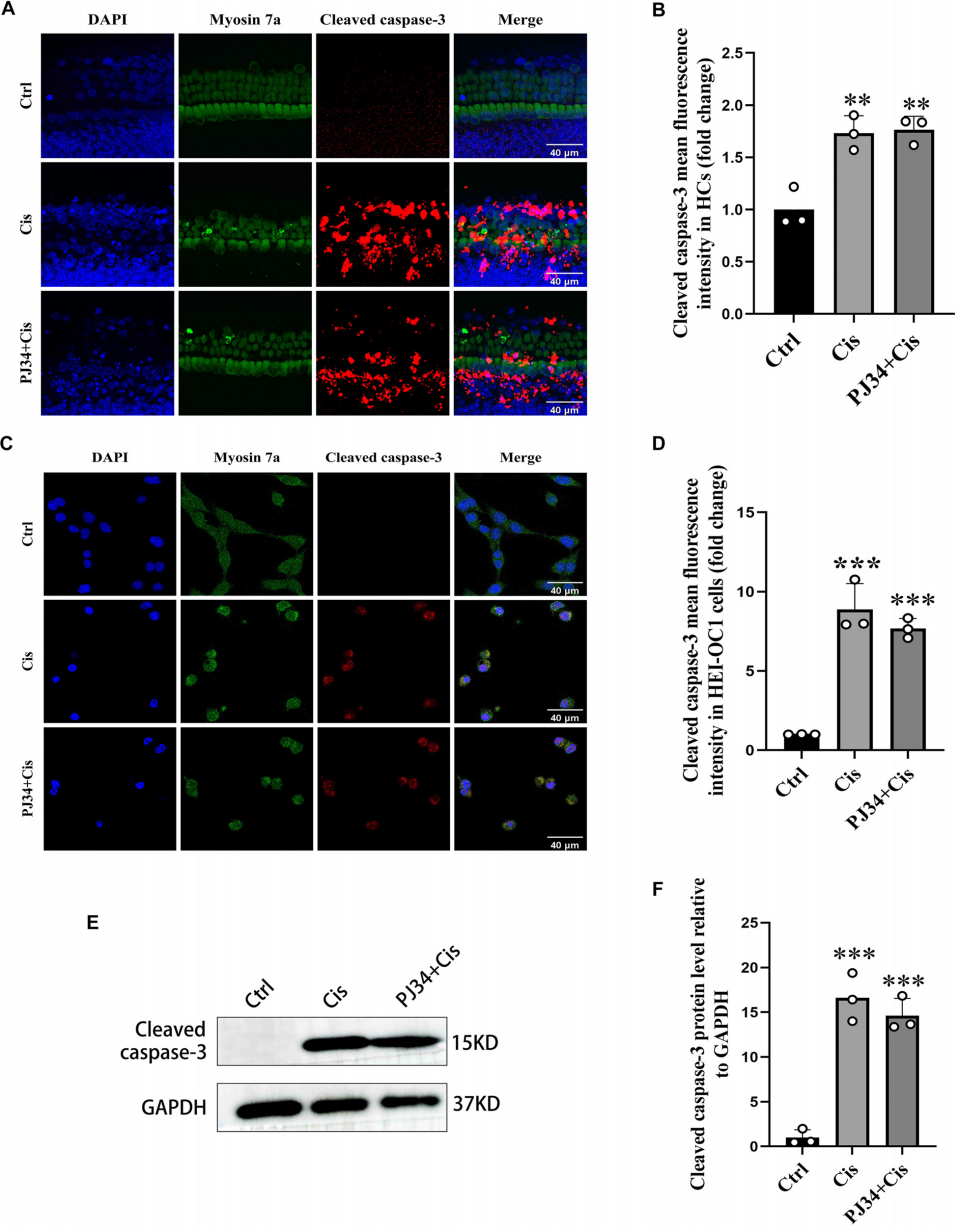
**PJ34 attenuates cisplatin-induced cell death in HEI-OC1 cells and HCs via a caspase-3–independent pathway.** (A) Double immunofluorescence staining of cleaved caspase-3 (red) and Myosin 7a (green) in control, cisplatin-treated, and PJ34 plus cisplatin-treated groups. (B) Quantitative analysis of cleaved caspase-3 fluorescence intensity shown in (A). Although cleaved caspase-3 expression increased significantly following treatment, there was no significant difference in fluorescence intensity between the treatment groups. (D) Quantitative analysis of cleaved caspase-3 levels shown in (C). HEI-OC1 cells exhibited trends similar to those observed in HCs. (E) Western blot analysis of cleaved caspase-3 protein levels in HEI-OC1 cells from control, cisplatin-treated, and PJ34 plus cisplatin-treated groups. GAPDH was used as a loading control. (F) Quantification of protein expression shown in (E). **P< 0.05 vs control group, ***P< 0.001 vs control group. *n* ═ 3. Scale bars ═ 40 µm. HCs: Hair cells.

Immunofluorescence analysis showed strong cleaved caspase-3 positivity in both HEI-OC1 cells and HCs in the cisplatin and PJ34 plus cisplatin groups (Figure 3A and 3C). Quantification of mean fluorescence intensity revealed no significant difference between these two groups (F(2,6) ═ 53.75; 0.87 ± 0.09 for Cisplatin in HEI-OC1; *P* > 0.05 and F(2,6) ═ 21.12; 1.02 ± 0.05 for Cisplatin in HCs; *P* > 0.05) (Figure 3B and 3D).

WB results showed a significant increase in cleaved caspase-3 protein levels in both the cisplatin group (F(2,6) ═ 55.44; 27.87 ± 18.28 for Control; *P* < 0.001) and PJ34 plus cisplatin group (23.97 ± 11.05 for Control, *P* < 0.001) compared to controls. However, no significant difference was observed between the two treatment groups (0.89 ± 0.07 for Cisplatin, *P* < 0.05) (Figure 3E and 3F).

In summary, both immunofluorescence and WB results indicate that PJ34 does not alter cleaved caspase-3 expression after cisplatin treatment, suggesting a caspase-independent mechanism.

### PJ34 attenuated PARP-1 hyperactivation after cisplatin exposure

**Figure 4.**
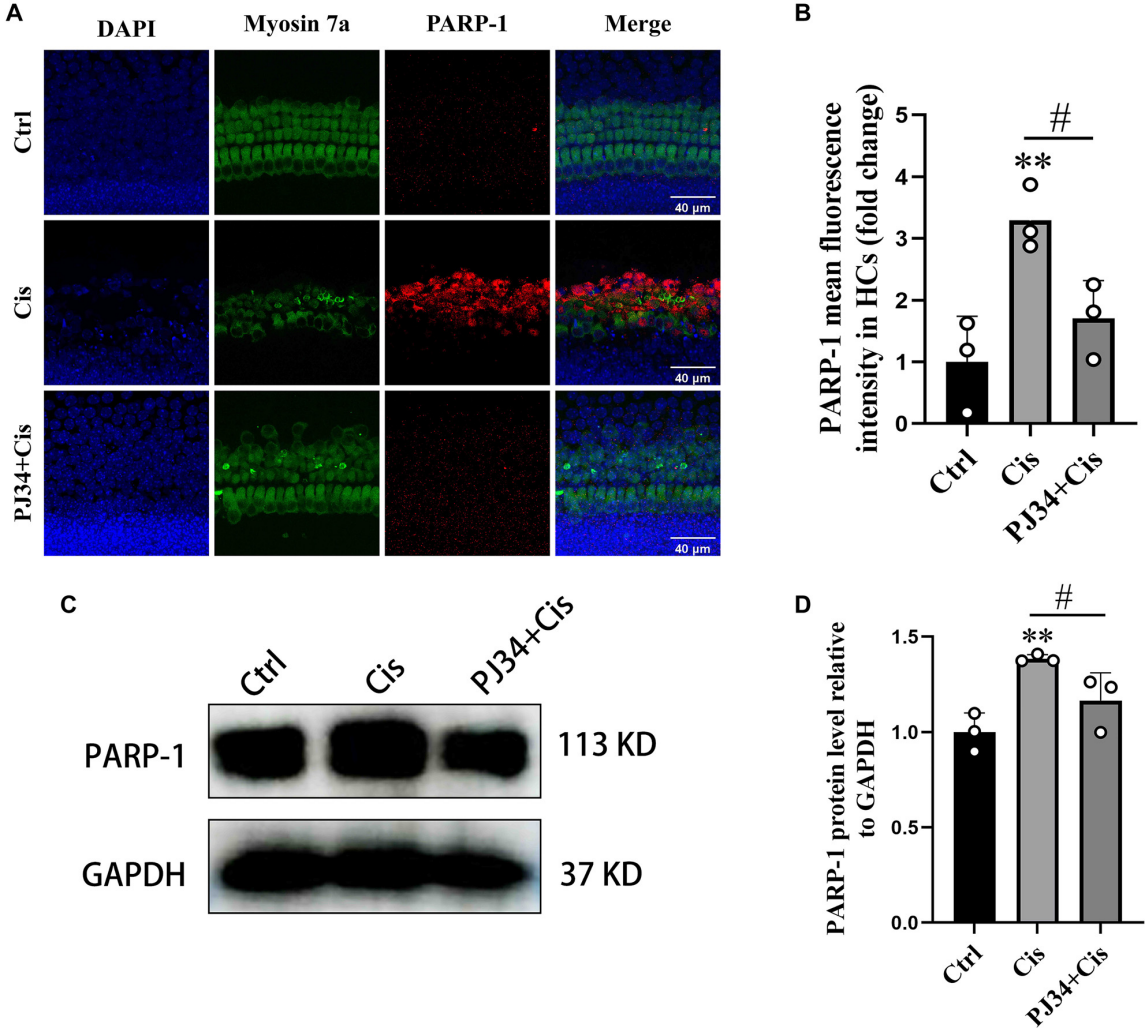
**PJ34 attenuates cisplatin-induced activation of PARP-1.** (A) Immunofluorescence showing changes in PARP-1 expression across different treatment groups; (B) Quantitative analysis of PARP-1 expression shown in (A); (C) Western blot analysis of dynamic changes in PARP-1 protein levels in HEI-OC1 cells following cisplatin exposure; (D) Quantitative analysis of PARP-1 expression shown in (C). *P< 0.05 vs control group, **P< 0.01 vs control group, ^#^*P*< 0.05 vs cisplatin group. PARP-1: Poly (ADP-ribose) polymerase-1.

Immunofluorescence showed stronger PARP-1 fluorescence in the cisplatin group compared to controls (F(2,6) ═ 10.34; 7.05 ± 7.76 for Control; *P* < 0.01), while PJ34 co-treatment reduced PARP-1 intensity (0.51 ± 0.19 for Cisplatin; *P* < 0.05) (Figure 4A and 4B).

WB confirmed a significant reduction in PARP-1 protein levels in the PJ34 plus cisplatin group compared to the cisplatin group (F(2,6) ═ 10.63; 0.84 ± 0.10 for Cisplatin; *P* < 0.05) (Figure 4C and 4D).

### Cisplatin induces cell death via AIF nuclear translocation in a caspase-independent manner, attenuated by PJ34 co-treatment

**Figure 5.**
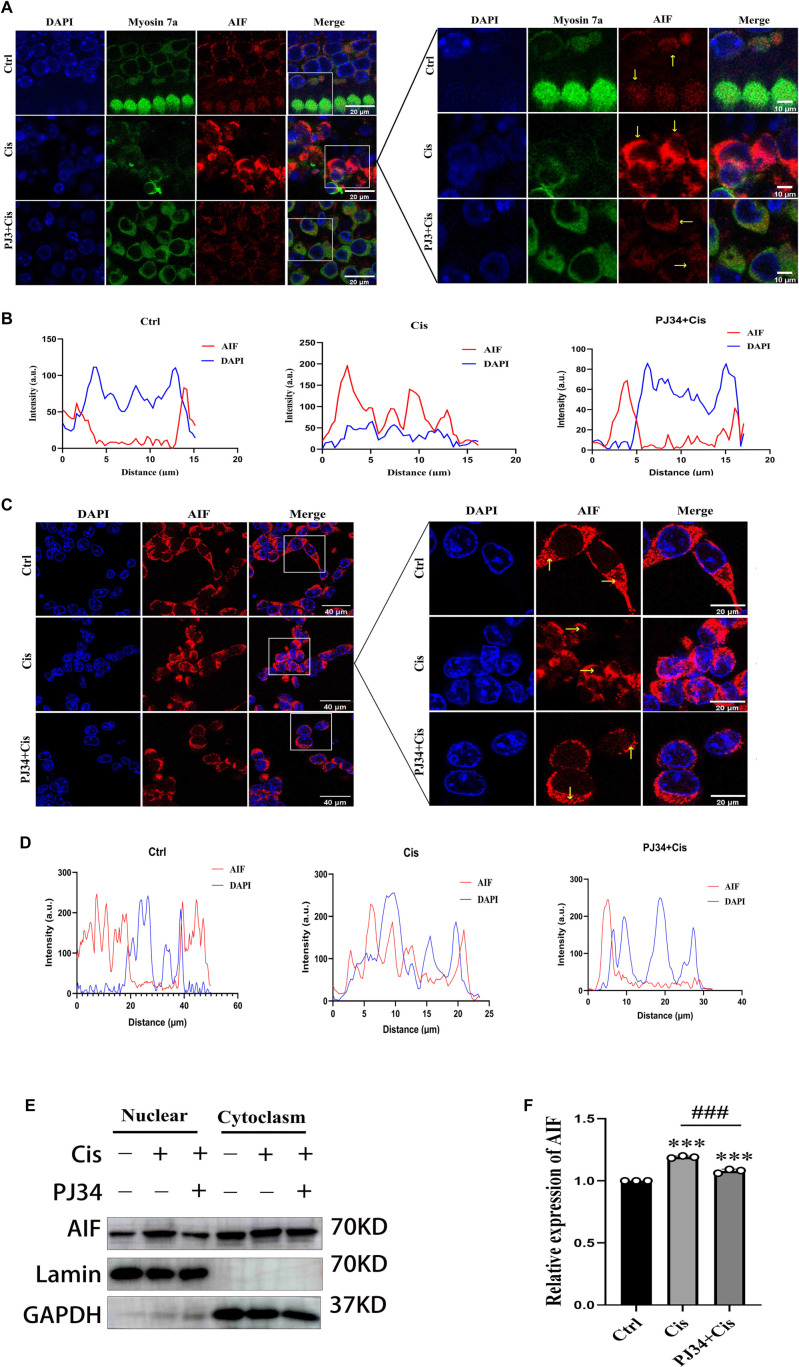
**Cisplatin promotes nuclear translocation of AIF in HCs and HEI-OC1 cells, which is attenuated by PJ34.** (A) Double immunofluorescence staining of AIF (red) and Myosin 7a (green) in control, cisplatin-treated, and PJ34 plus cisplatin-treated groups. The cytoplasm of HCs is labeled with Myosin 7a, and nuclei are labeled with DAPI (blue); (B) Co-localization analysis of AIF and DAPI in (A); (C) AIF localization in HEI-OC1 cells under different treatment conditions; (D) Co-localization analysis of AIF and DAPI in (C); (E) Cytoplasmic and nuclear proteins from HEI-OC1 cells were isolated using a nucleoplasmic separation kit, and lysates were analyzed by Western blot. Anti-lamin and anti-GAPDH antibodies were used as loading controls for nuclear and cytoplasmic fractions, respectively; (F) Quantitative analysis of the Western blot results shown in (E). ***< 0.001 vs control group. ^###^< 0.001 vs cisplatin group. HCs: Hair cell; AIF: Apoptosis-inducing factor.

Immunofluorescence of HCs revealed that in the control group, AIF was localized mainly in the cytoplasm (yellow arrows), with minimal nuclear fluorescence (Figure 5A). In the cisplatin group, HCs showed wrinkled cytoplasm and partial nuclear translocation of AIF. PJ34 co-treatment preserved cytoplasmic morphology, maintained AIF localization in the cytosol, and reduced nuclear translocation. Co-localization analysis confirmed that PJ34 mitigated cisplatin-induced AIF nuclear entry. Similar findings were observed in HEI-OC1 cells (Figure 5C).

Nucleocytoplasmic fractionation of HEI-OC1 cells showed increased nuclear AIF in the cisplatin group (F(2,6) ═ 255.4; 1.20 ± 0.008 for Control; *P* < 0.001), while PJ34 co-treatment reduced nuclear AIF levels (0.90 ± 0.02 for Cisplatin; *P* < 0.001) (Figure 5E and 5F).

### PJ34 alleviates cisplatin-induced MMP loss and mitochondrial ROS accumulation in HEI-OC1 cells and HCs

**Figure 6.**
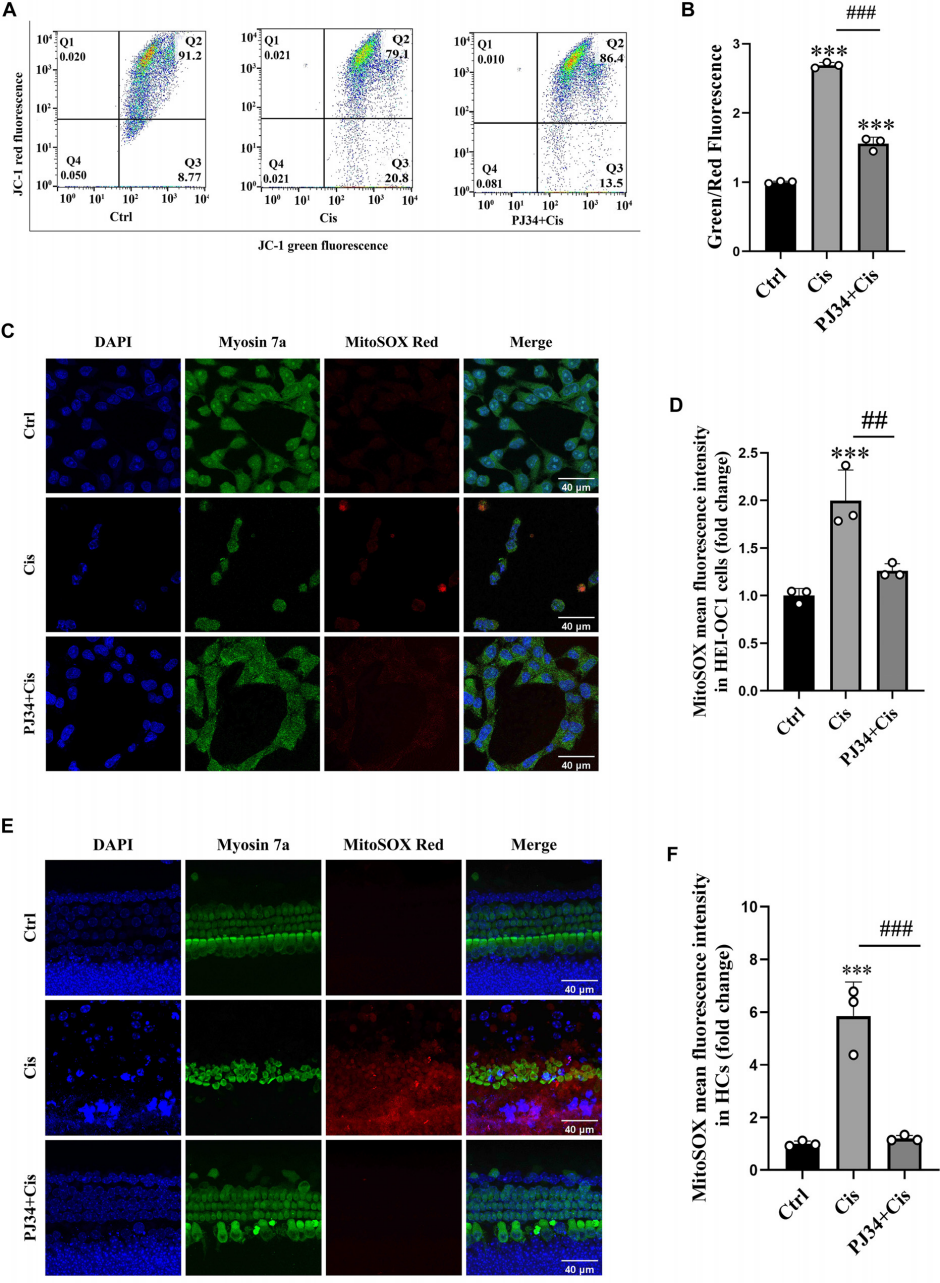
**PJ34 attenuates cisplatin-induced MMP loss and mitochondrial ROS increase in HEI-OC1 cells and HCs.** (A) Flow cytometry analysis of MMP in HEI-OC1 cells following treatment with cisplatin and/or PJ34; (B) Quantitative analysis of MMP data shown in (A); (C) Representative images of MitoSOX staining (red) in control, cisplatin, and PJ34 plus cisplatin groups. Myosin 7a (green) was used to label HEI-OC1 cells. Scale bars ═ 40 µm; (D) Quantitative analysis of mitochondrial ROS levels from (C), showing reduced ROS in PJ34-pretreated HEI-OC1 cells after cisplatin exposure; (E) Mitochondrial ROS levels in HCs showed a similar trend; (F) Quantitative analysis of mitochondrial ROS data shown in (E). ***P< 0.001 vs control group, ^##^*P*< 0.01 vs cisplatin group, ^###^*P*< 0.001 vs cisplatin group. *n* ═3. MMP: Mitochondrial membrane potential; HCs: Hair cells; ROS: Reactive oxygen species.

MitoSOX Red staining showed strong positivity after cisplatin action (F(2, 6) ═ 21.14, 2.02 ± 0.49, *P*< 0.001 for Control in HEI-OC1 cells, F(2, 6) ═ 40.06, 5.88 ± 1.44, *P* < 0.001 for Ctrl in HCs) and weaker positivity in the PJ34 plus cisplatin group (0.64 ± 0.06, *P* < 0.01 for Cisplatin in HEI-OC1 cells, 0.22 ± 0.05, *P* < 0.001 for Cisplatin in HCs ) in both HEI-OC1 cells and HCs (Figure 5C–5F). MMP assay exhibited a decrease in MMP due to cisplatin exposure compared to normal controls (F (2, 6) ═ 622.2, 2.70 ± 0.05 for Control, *P* < 0.001). Co-treatment with PJ34 alleviated the extent of MMP decline (0.58 ± 0.03 for Cisplatin, *P* < 0.001) (Figure 6A and 6B). These findings suggest that in cochlear HCs, cisplatin-induced injury elevates mitochondrial ROS and decreases MMP, while PJ34 co-treatment mitigates both effects.

## Discussion

In our current investigation, immunofluorescence staining demonstrated that the cochlear basement membrane exposed to cisplatin displayed considerable cell loss, along with morphological alterations and structural disorganization. The histologic changes in cisplatin-induced hearing loss are consistent with our prior study [19]. The results of flow cytometry and immunofluorescence showed that 2.5 µM PJ34 effectively reduced cisplatin-induced HEI-OC1 cell death. We then explored the influence of PJ34 on the anti-ovarian cancer effects of cisplatin. The outcomes showed an increase in apoptosis in all PJ34 co-treated cells compared to the cisplatin group, suggesting that PARPi increases the vulnerability of ovarian cancer cells to cisplatin, which aligns with previous findings [10, 28].

Subsequently, we investigated how PJ34 mitigates cisplatin-induced cellular damage. Our observations in the present study showed an increase in cleaved caspase-3 levels after cisplatin treatment, demonstrating that cisplatin initiates apoptosis mainly through the mitochondrial apoptotic pathway, which agrees with our previous study [26, 28]. We observed no significant change in cleaved caspase-3 expression with PJ34 co-treatment, indicating that PJ34 mitigated cisplatin-induced cell death in a caspase-independent manner. Previous reports have highlighted parthanatos as a newly identified death pathway in cochlear HCs, mediating noise-induced hearing loss [29, 30]. Thus, we postulated that parthanatos plays an important role in the mechanism by which PJ34 attenuates cisplatin-induced ototoxicity.

However, since both apoptosis and parthanatos involve the translocation of phosphatidylserine to the cell membrane surface, standard assay kits using only Annexin V and propidium iodide staining cannot distinguish apoptosis from parthanatos [31]. To address this challenge, we focused on key parthanatos events, specifically the activation of PARP-1 and changes in the subcellular localization of AIF. First, we investigated how cisplatin affects PARP-1 protein levels. The findings revealed an increase in PARP-1 expression following cisplatin exposure. In contrast, PJ34 effectively attenuated the cisplatin-induced upregulation of PARP-1. By pharmacologically inhibiting PARP-1, we demonstrated that it serves as a pivotal mediator of cisplatin-induced damage to HCs, with its activation closely associated with cisplatin-induced hearing loss. Inhibiting its expression may offer a promising strategy to mitigate cisplatin-induced ototoxicity.

Next, we examined the differences in AIF expression in the nucleus and cytoplasm after cisplatin and PJ34 treatments. As a mitochondrial protein, AIF’s pro-apoptotic function is attributed to its nuclear translocation. However, the mechanism of PARP-1-dependent AIF release from mitochondria remains unclear and may involve factors such as PAR generation, disruption of MMP, or mitochondrial fission [32]. Our results suggest that cisplatin promotes AIF translocation from the cytoplasm into the nucleus, where it collaborates with the caspase-dependent pathway to induce cell death. Co-treatment with PJ34 reduced nuclear AIF levels and attenuated cisplatin-induced cellular damage. Based on these findings, we propose that PARP-1- and AIF-mediated parthanatos plays a key role in cisplatin-induced hearing impairment. Furthermore, rescuing cells from parthanatos-dependent cell death may represent a key mechanism underlying PJ34’s hearing-protective effects.

Given the close association between parthanatos and mitochondrial dysfunction, we also explored changes in ROS levels and MMP after cisplatin treatment. MitoSOX staining showed that cisplatin increased mitochondrial ROS accumulation, while MMP assays indicated that cisplatin caused MMP loss. These findings suggest that ROS accumulation and MMP reduction are key contributors to cochlear HC injury. Additionally, PJ34 co-treatment attenuated both the ROS increase and MMP decrease, suggesting that PJ34 may alleviate mitochondrial dysfunction to reduce cisplatin-induced ototoxicity.

Notably, when exploring the optimal concentration of PJ34 to attenuate cisplatin-induced auditory cell damage, we found that its protective effect was gradually weakened as the concentration increased. This suggests a strict concentration threshold for PJ34’s otoprotective effect and that high concentrations may actually be detrimental to cochlear HCs. As a DNA repair inhibitor, high PJ34 doses may exacerbate DNA damage and promote apoptosis. Therefore, maximizing PARP-1 inhibition while minimizing toxicity is critical. Indeed, low doses of PJ34 were sufficiently protective in other non-tumor pathology models [6, 33]. Based on safety and efficacy, we prioritized the lower dose range during the pre-experimental phase.

Overall, one plausible interpretation of these results is that PJ34 may exhibit a dose-dependent inhibitory effect on DNA repair in the cochlea. This could lead to an imbalance in the DNA damage-repair system when PJ34 accumulation exceeds a certain threshold, potentially worsening cisplatin-induced HC damage. Moreover, the blood-labyrinth barrier (BLB)—a functional barrier composed of tightly connected, non-porous capillaries—limits drug transfer from the bloodstream to the inner ear [34]. Thus, when high doses of PJ34 are used as anticancer therapy, its concentration in the cochlea may remain low, avoiding toxicity to HCs. This underscores the importance of accurately determining dose-effect curves when combining drugs and strictly monitoring PJ34 concentrations to optimize their combined effects.

Although our study found that low concentrations of PJ34 conferred resistance to cisplatin ototoxicity, the mechanisms underlying different cumulative effects at varying concentrations remain unclear and warrant further investigation. Another possible explanation is the off-target effects of high PJ34 concentrations. Madison et al. reported that micromolar PJ34 levels induce mitotic arrest in cancer cells and fibroblasts, independent of PARP-1/2. Antolín et al. noted that PJ34 interacts with other targets, such as Pim1, which may cause cell cycle arrest at concentrations above 5 µM [35, 36]. Therefore, off-target effects, including Pim1 inhibition, should be considered when using PJ34. Co-treatment with cisplatin and 2.5 µM, 5 µM, or 10 µM PJ34 showed notable differences in cell survival, though all concentrations attenuated cisplatin-induced cochlear HC damage. This suggests a potential inflection point in the 2.5–5 µM range where PJ34 most effectively reduces cisplatin-induced ototoxicity and possibly exerts PARP-1-independent effects. To address this, it will be necessary to specifically inhibit Pim targets in HEI-OC1 cells and HCs to clarify their relationship to cell growth inhibition and develop strategies to minimize PJ34’s off-target effects.

Another interesting observation was that PJ34 did not significantly enhance the anticancer effect of cisplatin in TOV112D cell lines. One possible explanation is that the treatment concentration or duration of PJ34 was not optimal for sensitizing TOV112D cells to cisplatin. Alternatively, the 30 µM cisplatin concentration used may not have been ideal for inducing antitumor effects, as shown by only a marginal increase in apoptosis when cisplatin was used alone. Effective sensitization may require a higher cisplatin dose to cause sufficient DNA damage. Additionally, cisplatin resistance in TOV112D cells may result from genetic mutations or epigenetic changes reactivating BRCA1/2, thus restoring homologous recombination repair (HRR) [37]. Activation of non-homologous end-joining or other alternate repair pathways that bypass PARPi-mediated inhibition may also contribute to resistance [38]. These mechanisms require further investigation.

In this study, we demonstrated that PJ34 protects against cisplatin-induced cellular damage by inhibiting PARP-1–AIF-mediated parthanatos and reducing mitochondrial dysfunction. We also confirmed PJ34’s antitumor activity. Since PARPi were first used clinically to treat BRCA-mutant cancers, they have been widely adopted as monotherapies or in combination with platinum-based drugs for ovarian and breast cancers [39]. Our findings show for the first time that PJ34 attenuates cisplatin-induced damage to HEI-OC1 cells and HCs without compromising cisplatin’s antitumor efficacy—representing the main innovation and value of this study compared to others. A key challenge in studying cisplatin otoprotective agents is ensuring they do not interfere with cisplatin’s anticancer effects. Many drugs thought to protect auditory cells have been found to reduce cisplatin efficacy [40]. The dual effects of PJ34 in reducing both cisplatin ototoxicity and nephrotoxicity, while preserving antitumor function, make it a promising candidate among otoprotective agents—representing a significant advancement in audiology.

Nonetheless, this study has some limitations. Our experiments showed that cisplatin treatment for 24 h elevated ROS and activated PARP-1. However, the 24-h window did not allow us to determine whether one event precedes the other. Evidence suggests a feedback loop where ROS accumulation triggers PARP-1 overexpression, which in turn increases ROS production, ultimately driving PARP-1-mediated parthanatos [3]. Whether such a relationship exists in cisplatin-induced auditory cell damage remains to be confirmed.

Secondly, while we showed that PJ34 reduced ROS accumulation and MMP loss after cisplatin treatment, the relationship between ROS generation and MMP has not been explored. Likewise, the link between ROS accumulation and AIF nucleocytoplasmic relocalization remains unclear. While most studies suggest that ROS promotes AIF translocation via mitochondrial damage (e.g., MMP loss, permeability transition pore opening, and calcium overload) [41–43], these conclusions often rely on ROS scavengers like NAC. Future studies using specific AIF translocation inhibitors could clarify this mechanism. Furthermore, how PJ34-mediated reduction in DNA damage attenuates AIF translocation remains unknown. Although AIF translocation is linked to DNA fragmentation [44, 45], excessive DNA damage may overactivate PARP-1 and increase AIF nuclear translocation—a mechanism requiring further investigation.

Lastly, our study was limited to PJ34 and cisplatin effects in HEI-OC1 cells and P3 mouse cochlear explants. As this was an *in vitro* study, without *in vivo* validation in adult mice, the findings remain theoretical. Conducting *in vivo* experiments is a key next step toward clinical application.

## Conclusion

In summary, the results of the current study demonstrate that cisplatin may induce the death of HEI-OC1 cells and HCs through both caspase-dependent and caspase-independent pathways. In addition, PJ34 confers protective effects on cochlear HCs against cisplatin-induced damage by suppressing parthanatos. Crucially, PJ34 may offer a practical otoprotective strategy for patients receiving cisplatin. This discovery is particularly exciting given the severe ototoxicity associated with cisplatin, and PJ34 holds great promise for use in combination with platinum-based agents in the treatment of various malignant tumors. Further studies are needed to better understand the specific mechanisms by which PJ34 mitigates cisplatin-induced ototoxicity.

## Data Availability

The data that support the findings of this study are openly available in Mendeley Data at https://data.mendeley.com/datasets/zxzgp65bx5/1. All other data presented are contained within the manuscript/supplemental data.
